# Diagnostic accuracy of a commercially available deep-learning algorithm in supine chest radiographs following trauma

**DOI:** 10.1259/bjr.20210979

**Published:** 2022-03-24

**Authors:** Jacob Gipson, Victor Tang, Jarrel Seah, Helen Kavnoudias, Adil Zia, Robin Lee, Biswadev Mitra, Warren Clements

**Affiliations:** Department of Radiology, Alfred Health, Melbourne, Victoria, Australia; Department of Radiology, Alfred Health, Melbourne, Victoria, Australia; Faculty of Medicine, University of Queensland, Brisbane, Queensland, Australia; Department of Radiology, Alfred Health, Melbourne, Victoria, Australia; Harrison.ai, Sydney, NSW, Australia; Department of Radiology, Alfred Health, Melbourne, Victoria, Australia; Department of Surgery, Monash University, Melbourne, Victoria, Australia; Department of Radiology, Alfred Health, Melbourne, Victoria, Australia; Department of Radiology, Alfred Health, Melbourne, Victoria, Australia; National Trauma Research Institute, Melbourne, Victoria, Australia; Emergency & Trauma Centre, The Alfred Hospital, Melbourne, Victoria, Australia; School of Public Health & Preventive Medicine, Monash University, Melbourne, Victoria, Australia; Department of Radiology, Alfred Health, Melbourne, Victoria, Australia; Department of Surgery, Monash University, Melbourne, Victoria, Australia; National Trauma Research Institute, Melbourne, Victoria, Australia

## Abstract

**Objectives:**

Trauma chest radiographs may contain subtle and time-critical pathology. Artificial intelligence (AI) may aid in accurate reporting, timely identification and worklist prioritisation. However, few AI programs have been externally validated. This study aimed to evaluate the performance of a commercially available deep convolutional neural network – Annalise CXR V1.2 (Annalise.ai) – for detection of traumatic injuries on supine chest radiographs.

**Methods:**

Chest radiographs with a CT performed within 24 h in the setting of trauma were retrospectively identified at a level one adult trauma centre between January 2009 and June 2019. Annalise.ai assessment of the chest radiograph was compared to the radiologist report of the chest radiograph. Contemporaneous CT report was taken as the ground truth. Agreement with CT was measured using Cohen’s κ and sensitivity/specificity for both AI and radiologists were calculated.

**Results:**

There were 1404 cases identified with a median age of 52 (IQR 33–69) years, 949 males. AI demonstrated superior performance compared to radiologists in identifying pneumothorax (*p* = 0.007) and segmental collapse (*p* = 0.012) on chest radiograph. Radiologists performed better than AI for clavicle fracture (*p* = 0.002), humerus fracture (*p* < 0.0015) and scapula fracture (*p* = 0.014). No statistical difference was found for identification of rib fractures and pneumomediastinum.

**Conclusion:**

The evaluated AI performed comparably to radiologists in interpreting chest radiographs. Further evaluation of this AI program has the potential to enable it to be safely incorporated in clinical processes.

**Advances in knowledge:**

Clinically useful AI programs represent promising decision support tools.

## Introduction

The initial chest radiograph of patients after trauma may contain time critical pathology requiring rapid diagnosis and is routinely performed during trauma reception and resuscitation.^1,2^ Findings are often subtle and may be missed. With increasing workloads and limited staff outside of normal working hours, radiologists often prioritise reporting emergency cross-sectional imaging over plain radiographs. This leads to delays between image acquisition and reporting, and potentially to delays in diagnosis and treatment.

One method to overcome these problems is with the use of artificial intelligence (AI), which could provide a near instantaneous report to the practitioner at the bedside or flag potential abnormal studies to a radiologist for prioritised reporting. In developing countries, similar AI could provide access to expertise, which may not otherwise be available without telehealth services.

Previous studies have reported diagnostic accuracy of AI equivalent to that of physicians in visual recognition tasks such as identifying pulmonary embolism,^3^ stroke,^4^ skin cancer^5^ and diabetic retinopathy.^6^ However, real-world performance of AI is highly dependent on the training dataset. AI may perform effectively when presented with similar data to that it was trained on, but have poor generalisability when exposed to images obtained in new settings due to differences in hardware, exposure and technique. It is therefore conceivable that real-world performance of AI may be worse than that presented in initial validation studies. Despite this, a 2019 review reported only 6% of published AI algorithms are externally validated.^7^

The aim of this study was to independently validate the performance of an AI algorithm in detecting traumatic injuries on supine AP chest radiographs, and compare this to the accuracy of radiologist interpretation of the same chest radiograph, using contemporaneous CT as the ground truth.

## Methods and materials

### Setting

The Victorian state trauma system mandates that major trauma patients are transported directly to a trauma centre within 45 min transport time from the scene, and that all patients receive definitive care at a major trauma service. This project was performed at The Alfred Hospital, a level one adult trauma and tertiary university hospital servicing the state of Victoria, Australia that receives major trauma patients aged 16 years and over.^8^

### Inclusion and exclusion criteria

This retrospective study identified all cases from January 2009 to June 2019 where patients who presented after blunt trauma underwent both chest radiograph and CT within 24 h of arrival to hospital, where the imaging report for the radiograph was provided before the CT was performed. To ensure a fair comparison with the algorithm, patients with prior imaging at the time of reporting of the chest radiograph were excluded. This ensured that the reporting radiologists did not have access to additional information such as prior reports that might improve their performance. Patients were identified through searching the Picture Archive and Communication System.

### Radiologist performance and ground truth

Reports of chest radiographs and chest CTs were reviewed by two radiology residents. The chest radiographs and CTs were reported on the same day as image acquisition by a consultant radiologist. Given the scans were acquired and reported in the clinical setting the reporting Doctor for the radiographs and CT were not necessarily the same. Additionally, given the constraints of clinical workflow, there was variable time lag between the radiograph report and the subsequent CT report. The presence of any of the target pathologies within the reports produced at the time of the study were recorded in spreadsheets. The target pathologies were pneumothorax, rib fracture, clavicle fracture, humerus fracture, scapular fracture, lobar/segmental collapse and pneumomediastinum.

Ground truth of the presence or absence of pathology was based on the report of the contemporaneous CT. Agreement with the ground truth label was measured separately for the radiologist chest radiograph report, and the AI output based on the chest radiograph. This was measured using Cohen’s κ.

**Figure 1.**
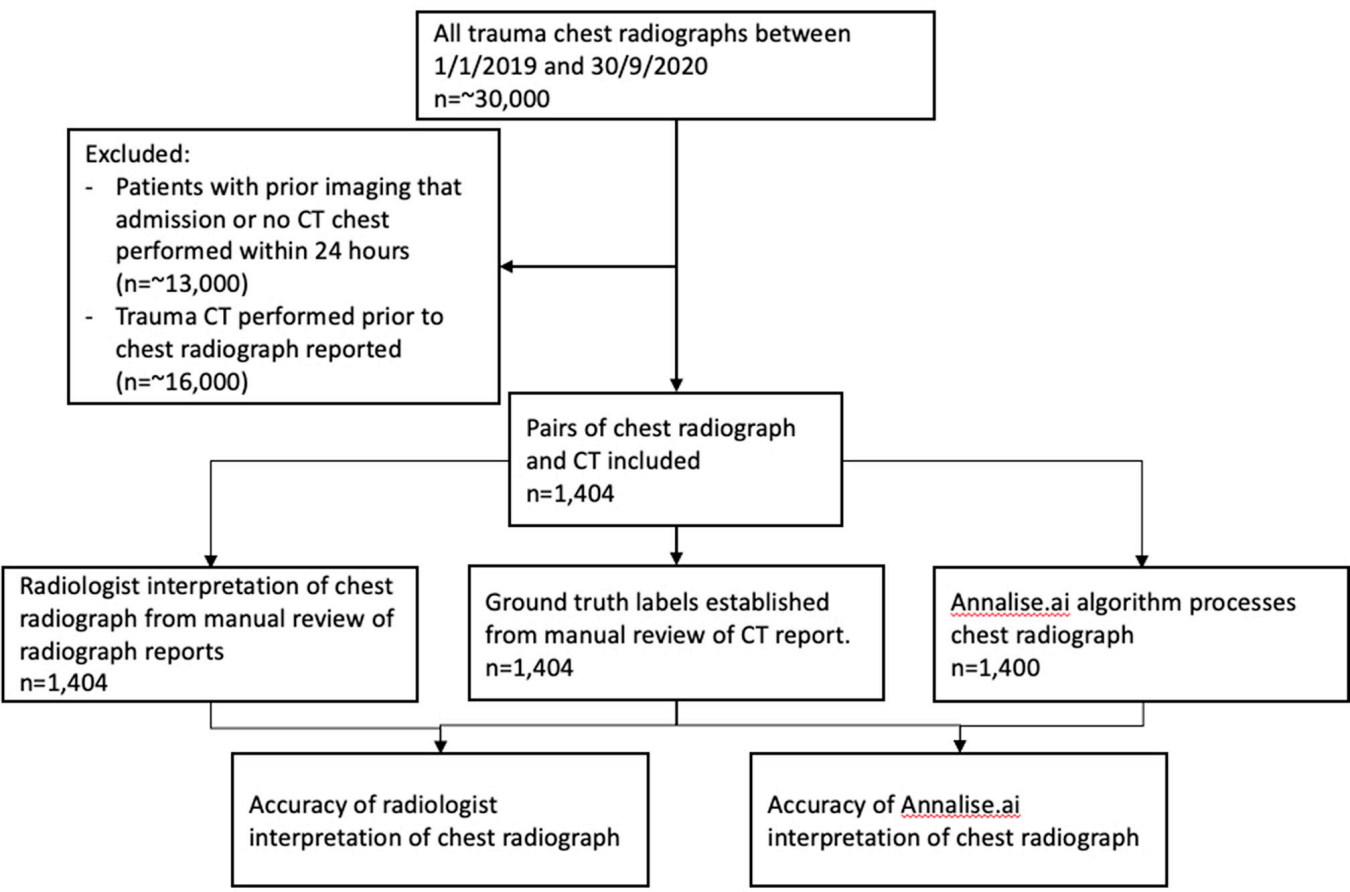
Patient flow diagram

Figure 1 demonstrates the patient flow diagram of this project.

### AI algorithm

The AI algorithm evaluated was version 1.2 of the Annalise CXR software provided by Annalise.ai^®^ (subsequently referred to as Annalise.ai). The AI is a commercially available deep-convolutional neural network trained on over 500,000 chest radiographs, which have been hand labelled by three radiologists each. It was downloaded to the institution’s servers and run retrospectively over anonymised DICOM data. The AI is trained to identify over 100 different pathologies and variants. For this study, we limited our examination to the findings most relevant in trauma.

### Image acquisition

Trauma chest radiographs are routinely obtained as a single supine AP view and are performed in the trauma centre. Almost all were obtained using a fixed Carestream^®^ DR Pinnacle X-ray machine. Chest CTs were obtained on a Toshiba^®^ One Acquillion and Toshiba One Acquillion Genesis 320 slice CTs with IV contrast unless contraindicated.

### Statistical testing

Baseline characteristics were summarised using median and interquartile ranges (IQR). Sensitivity, specificity and Cohen’s κ statistics were calculated using Microsoft Excel. Confidence intervals for sensitivity and specificity were calculated using the exact Clopper Pearson method using MedCalc statistical calculator (MedCalc Software, Ostend, Belgium). Statistical significance of difference in Cohen’s κ was estimated using a 10,000 iteration paired bootstrap to estimate the median, 2.5th percentile, 97.5th percentile of the difference in Cohen’s κ. *P*-values less than 0.05 were considered statistically significant.

### Proposed hybrid model

In our proposed workflow model the AI is integrated into the reporting workflow. This creates a “hybrid model” where a pathology was considered present if either the radiologist or AI identified it.

## Results

There were 1,404 pairs of chest radiographs and CTs from unique patients identified and available for analysis. 949 (68%) were male. The median age was 52 (IQR33-69) (Table 1). Four chest radiographs were technically inadequate and unable to be processed by the AI, leaving 1400 pairs included for Annalise.ai interpretation. Median time between radiograph and CT acquisition was 1.4 h (interquartile range 0.96–2.64 h).

**Table 1.** Characteristics of included cases

Age (years)	52 (IQR 33–69)
Sex	949 (68%) male
	455 (32%) female
Pathology on CT	**n (%)**
Pneumothorax	172 (12.3%)
Lobar/segmental lung collapse	37 (2.6%)
Pneumomediastinum	27 (1.9%)
Rib fracture	349 (24.9%)
Clavicle fracture	79 (5.6%)
Humerus fracture	31 (2.2%)
Scapula fracture	55 (3.9%)

Among included patients, 927 cases (66.0%) had no target pathologies on CT reports, 477 (34.0%) had at least one and 474 had more than one (33.4%). The most common pathology was acute rib fracture in 349 (24.9%), followed by pneumothorax in 172 (12.3%) (Table 1).

Agreement with CT as assessed using Cohen’s κ are listed in Table 2. This demonstrated statistically superior performance of AI compared to radiologists in detecting pneumothorax (*p* = 0.007) and lobar/segmental collapse (*p* = 0.012). Statistically inferior performance of AI in clavicle (*p* = 0.002), humerus (*p* < 0.001) and scapular fracture (*p* = 0.014). No statistically significant differences were demonstrated in assessing rib fracture and pneumomediastinum.

**Table 2.** [3] Agreement of radiologist radiograph report and Annalise.ai algorithm with CT report as measured using Cohen’s κ

Pathology	Radiologist agreement with CT	Annalise.ai agreement with CT	Paired Difference in Cohen’s κ	*p*-valuea
Rib fracture	0.40 (0.34–0.45)	0.39 (0.33–0.44)	−0.006 (−0.67–0.056)	0.210
Clavicle fracture	0.67 (0.57–0.77)	0.52 (0.42–0.62)	−0.152 (−0.263–−0.042)	0.002
Humerus fracture	0.77 (0.58–0.87)	0.38 (0.15–0.51)	−0.409 (−0.625–−0.194)	<0.001
Scapular fracture	0.38 (0.25–0.54)	0.23 (0.14–0.35)	−0.152 (−0.299–−0.001)	0.014
Pneumothorax	0.44 (0.36–0.52)	0.53 (0.45–0.60)	0.079 (0.006–0.153)	0.007
Pneumomediastinum	0.19 (0.00–0.39)	0.19 (0.00–0.39)	0.007 (−0.158–0.177)	0.200
Segmental collapse	0.19 (0.04–0.35)	0.36 (0.20–0.50)	0.165 (0.003–0.330)	0.012

a *P*-values calculated using a 10,000 iteration paired bootstrap method.

Both radiologists and Annalise.ai demonstrated high specificity of greater than 95% for all pathologies (Table 3). Sensitivity and specificity were also calculated if either the radiologist or AI identified a pathology, to simulate the potential effect of radiologists having access to AI when reporting. Sensitivity for all findings improved, with minor decreases in specificity. However, specificity remained >95% for all findings.

**Table 3.** Test characteristics of Annalise.ai interpretation compared to radiologist

	Radiologist reporting of CXR						Annalise.ai						AUC (95% CI)	Radiologist & Annalise.ai combined	
	TP	FP	TN	FN	Sensitivity	Specificity	TP	FP	TN	FN	Sensitivity	Specificity		Sensitivity Specificity	
**Pneumothorax**	57	8	1224	115	33.1% (26.2–40.7)	99.4% (98.7–99.7)	67	2	1227	104	39.2% (31.8–46.9)	99.8% (99.4–100)	0.926 (0.896–0.953)	45.6% (38.0–53.4)	99.2% (98.5–98.5-99.6)
**Pneumomediastinum**	3	1	1376	24	11.1% (2.4–29.2)	99.9% (99.6–100)	3	0	1373	24	11.1 (2.4–29.2)	100% (99.7–100)	0.872 (0.786–0.958)	14.8% (4.2–33.7)	99.9% (99.6–100)
**Rib fracture**	113	13	1042	236	32.4% (27.5–37.6)	98.8% (97.9–99.3)	143	75	977	205	41.1% (35.9–46.5)	92.9% (91.2–94.4)	0.749 (0.717–0.780)	49.4% (44.1–54.8)	91.9 (90.1–93.5)
**Clavicle fracture**	43	3	1322	36	54.4% (42.8–65.7)	99.8% (99.3–100)	44	37	1284	35	55.7% (44.1–66.9)	97.2% (96.2–98.0)	0.831 (0.775–0.887)	69.6% (58.3–79.5)	97.1 (96.0–97.9)
**Humerus fracture**	21	2	1371	10	67.7% (48.6–83.3)	99.9% (99.5–100)	10	8	1361	21	32.3% (16.7–51.4)	99.4% (98.9–99.8)	0.836 (0.743–0.929)	74.2% (55.4–88.1)	99.3% (98.8–99.7)
**Scapular fracture**	15	5	1344	40	27.3% (16.1–41.0)	99.6% (99.1–99.9)	19	64	1281	36	34.6% (22.2–48.9)	95.2% (94.0–96.3)	0.855 (0.790–0.920)	45.5% (32.0–59.5)	94.9 (93.6–96.1)
**Lobar/segmental collapse**	4	1	1366	33	10.8% (3.0–25.4)	99.9% (99.6–100)	13	21	1343	23	36.1% (20.8–53.8)	98.5% (97.7–99.0)	0.917 (0.856–0.979)	36.1% (20.8–53.8)	98.5% (97.7–99.0)

FN, false negative; FP, false positive; TP, true positive; TP, true positive.

**Figure 2.**
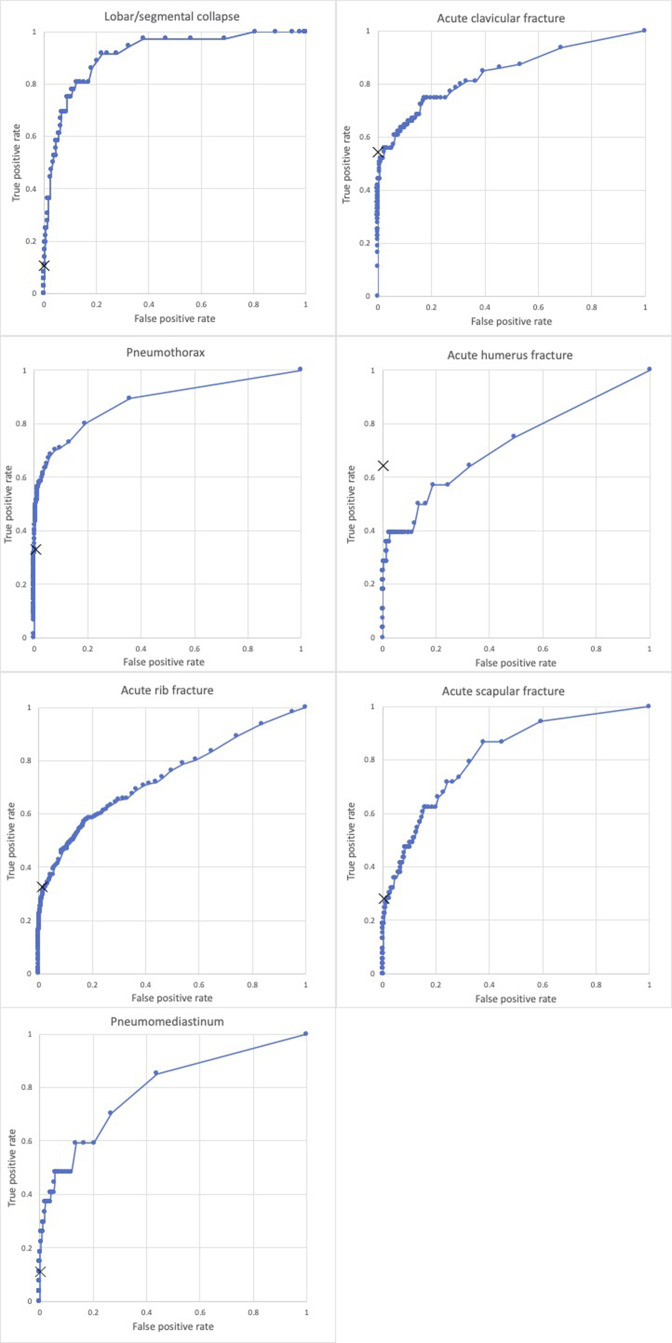
Receiver operating characteristic curves. Radiologist interpretation represented by cross, AI by line

**Figure 3.**
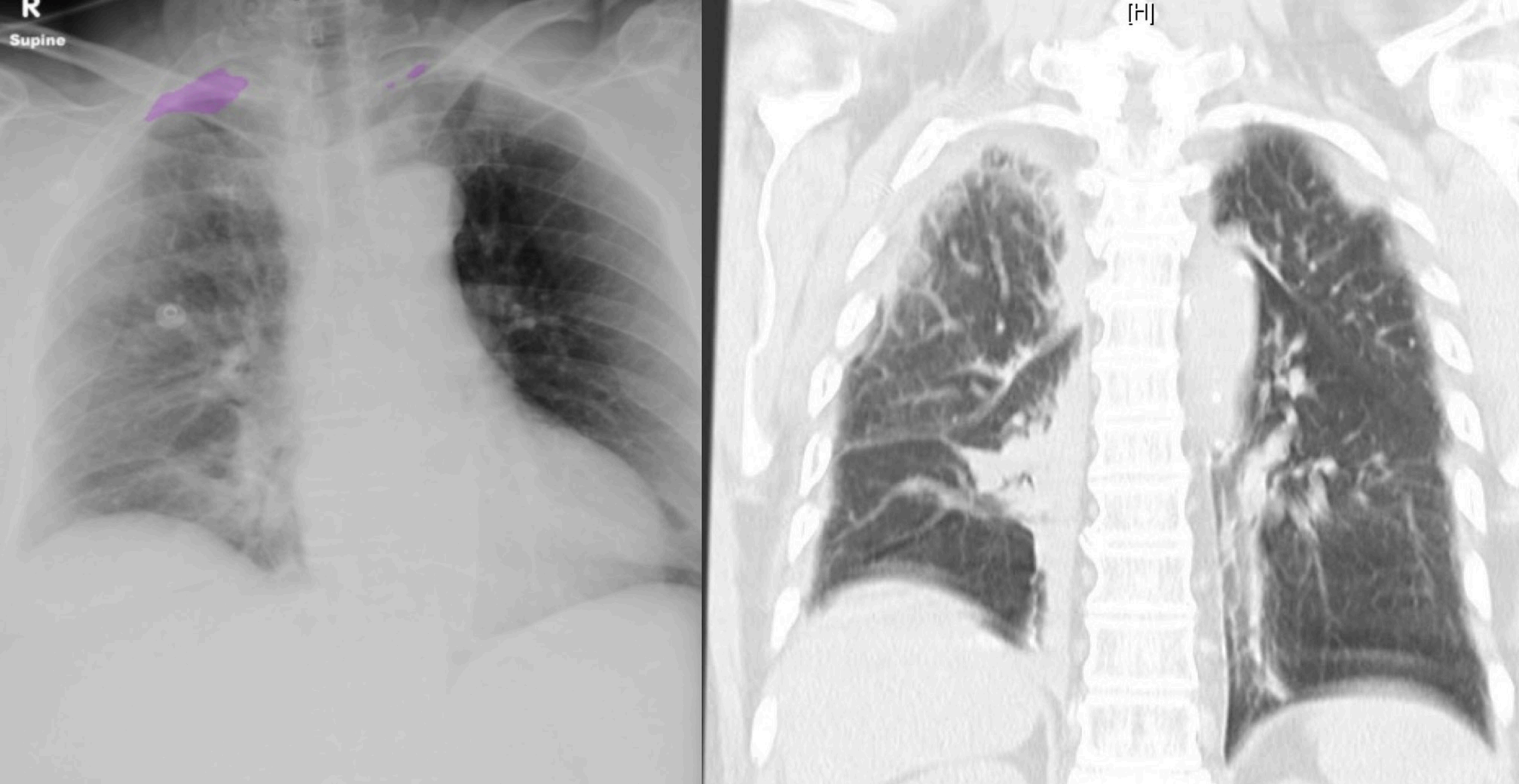
A case of false positive by the A.I., reported correctly by the radiologist. *A,* Supine chest radiograph in a 76 year old lady demonstrates multiple right-sided rib fractures but no definitive pneumothorax. The overlying AI segmentation maps incorrectly identify small biapical pneumothoraces. *B*, Corresponding CT coronal slice demonstrates partial right lower lobe collapse which could account for the change in radiodensity which the AI had mistaken for a pneumothorax.

Receiver operating curves for each category of pathology are demonstrated in Figure 2. Examples of the AI output available are shown in Figure 3.

## Discussion

Annalise.ai was superior or equivalent to radiologist performance in four of seven target pathologies, only performing inferiorly in identifying fractures of the shoulder girdle. Importantly, AI was superior to radiologists in detecting pneumothoraces and no statistical difference was found in identifying rib fractures, two of the most common pathologies in thoracic trauma.

CT is considered the gold standard for the diagnosis and detection of traumatic injury.^9^ It has high sensitivity and specificity for the detection of traumatic injury to the lung, pleura and chest wall.^10^ These qualities make chest CT the ideal ground truth.

AI demonstrated superior agreement with CT in detecting pneumothorax as measured with Cohen’s κ, although small individual differences in sensitivity and specificity between AI and radiologists were not statistically significant. This is reassuring as pneumothoraces may require urgent management, and conversely false-positive diagnoses may lead to unnecessary chest drain insertion. It is probable that most radiologist missed pneumothoraces were small and delays in diagnosis before CT unlikely to be clinically significant. Data on the size of pneumothoraces were not captured, and it is therefore unknown if this would be the case for pneumothoraces missed by AI.

**Figure 4.**
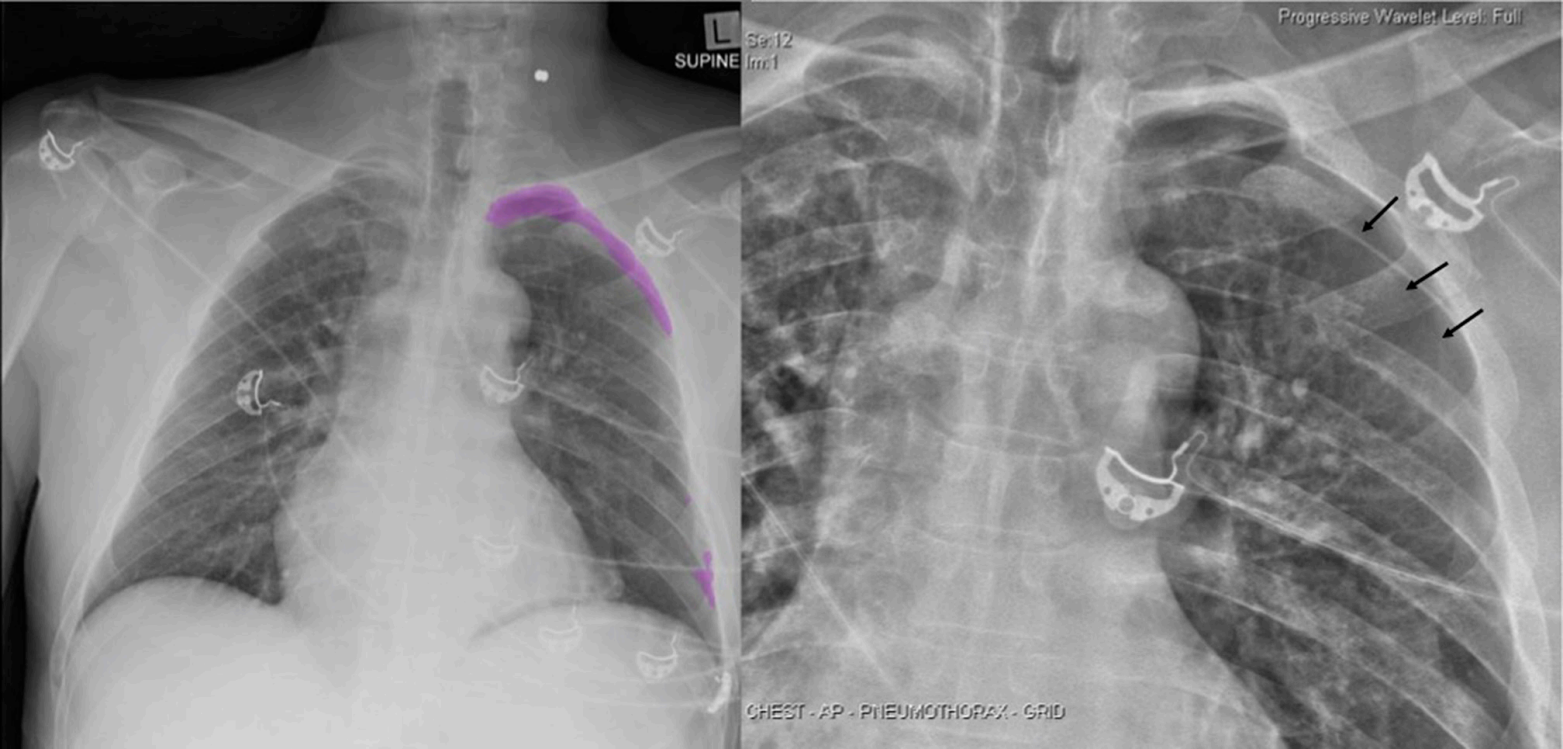
Left lateral pneumothorax missed by radiologist but identified by A.I. *A,* AI segmentation map correctly identifies the pneumothorax. *B,* Magnified view of the left hemithorax with texture-enhancement filter demonstrates a subtle but definite pleural edge (arrow). Minimally displaced left rib fractures are also noted.

**Figure 5.**
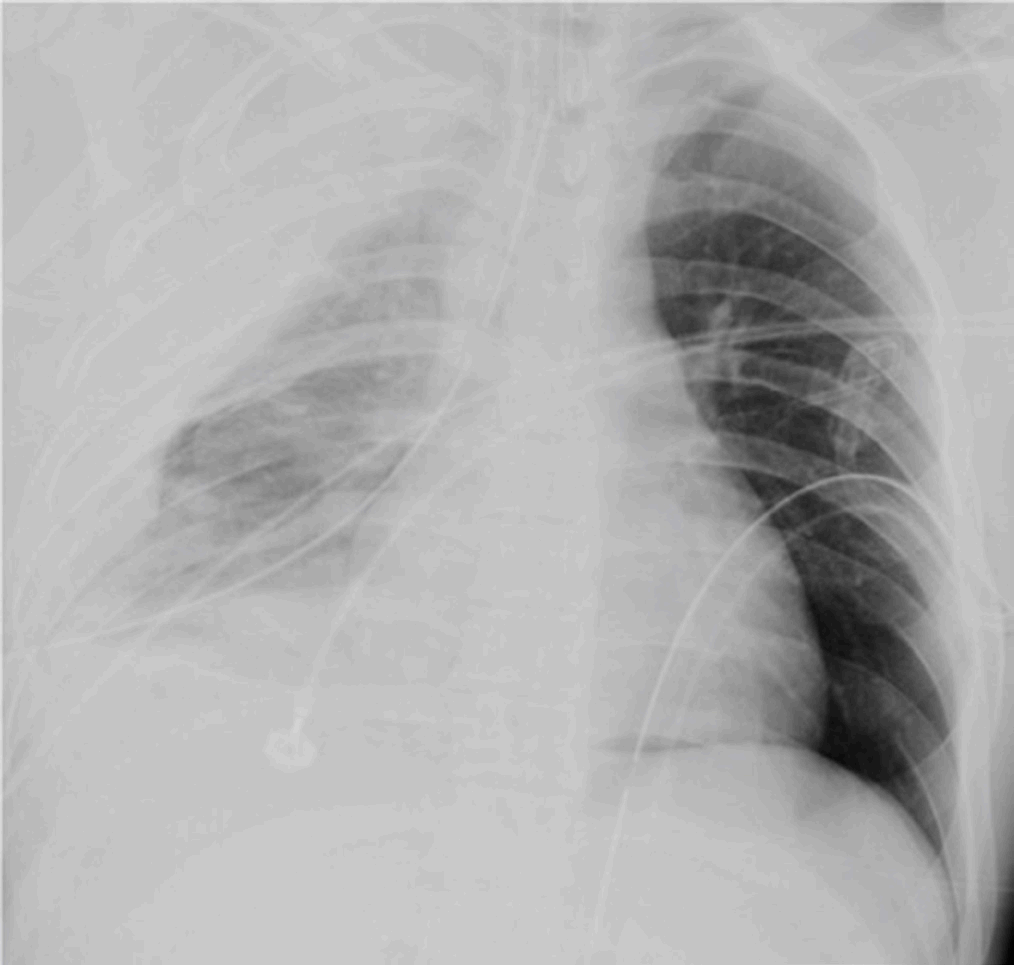
Small left anterior pneumothorax missed by AI but identified by radiologist

Review of AI generated segmentation maps may explain reasons for missed pneumothorax. Figure 3 is false-positive by the AI. In this example, an area of atelectasis, seen more clearly on CT, is mistaken as a pleural edge. Figure 4 demonstrates a pneumothorax missed by the radiologist but identified by AI. In retrospect, there is an obvious pneumothorax (Figure 4b). This highlights one benefit of AI is not in detecting pneumothoraces beyond the perception of humans, but in its resistance to human fallibilities such as fatigue or inattention which despite our best efforts occasionally lead to missed diagnoses such as this. A left pneumothorax presenting with a deep sulcus sign was missed by the AI but identified by the radiologist (Figure 5). This case highlights the importance of subgroup analysis of AI performance, in particular in regards to atypical pneumothorax presentation as an area of improvement for AI algorithms. The radiologist may also have relied on contextual clues such as the presence of an intercostal catheter in implying the presence of a pneumothorax.

AI also outperformed radiologists in diagnosing partial lung collapse, although this may be due to radiologists not considering this to be a relevant or important finding in the setting of trauma and so omitting this from their reports even when identified.

Radiologists outperformed AI in diagnosing clavicle, humerus and scapula fractures. One explanation for this is that radiologists may have had access to additional clinical information or other radiographs which would have informed their interpretation. This is unlike the AI which is unable to incorporate additional information or change its diagnosis based on clinical suspicion, and analyses only the chest radiograph.

The results of our ‘hybrid model’ showed increased sensitivity for every finding, although, sensitivity for all pathologies on CXR remained too low to be of clinical utility. An alternative model to consider would be allowing the radiologist to decide whether to accept the AI’s recommendation. In this model, the AI would have a complementary role as a second reader. This supports the findings of a previous study which demonstrated that when a similar AI was provided to residents, their performance at detecting fractures on radiographs approached the level of a specialist radiologist.^11^ These various models have been trialled in other radiological settings such as screening mammograms,^12^ and the ideal work-flow integrating AI requires further research.

### How AI compares to literature

One important way in which Annalise.ai differs from other AIs is its ability to identify multiple pathologies simultaneously. In particular, this AI will consider the possible correlation between the various findings. Using this correlation the AI can increase or decrease the likelihood of other findings. This is in contrast to most published AIs which assess for a single pathology, limiting their clinical utility and potentially exacerbating the perception of AI as being a binary program with limited real-world capabilities. Hwang et al^13^ previously assessed an algorithm which could classify multiple thoracic diseases, however it appears each radiograph only had one pathology. The capability of an AI to accurately detect multiple pathologies simultaneously is crucial as over 40% of trauma patients will present with multiple injuries.^14^

Although overall AI specificity was similar to radiologists, previous studies have reported significantly higher sensitivities of other AIs. A 2021 meta-analysis of ten studies demonstrated AI detection of pneumothorax on chest radiograph had a pooled sensitivity of 0.7 (95% CI 0.45–0.87).^15^ Some AIs have even reported sensitivities of up to 90% in diagnosing pneumothorax, significantly higher than that reported here, albeit at slightly lower specificities.^16,17^ This difference is likely attributable to methodological differences. In most previous studies the ground truth label was based on consensus or subspecialist radiologist interpretation of the same chest radiograph, rather than CT. The lower sensitivity described here may reflect the limitations of radiography, rather than AI. Supine positioning of our cohort may also have contributed to decreased sensitivity. A recently evaluated model assessing supine pneumothoraces similarly showed reduced sensitivity compared to other models evaluating erect radiographs.^18^

A prior deep-learning model for rib fracture detection demonstrated comparable performance to Annalise.ai, achieving a sensitivity of 44.2%.^19^ Noticeably this similar level of performance was achieved by Annalise.ai despite using CT as a reference standard compared to radiologist-consensus radiograph review, with CT being a significantly more accurate ground truth for rib fracture detection.^20^ An algorithm developed to identify clavicle fracture had a much higher sensitivity of 89.7%^21^; however, it is unclear from the methodology as to the radiographic views available to the AI. Possible dedicated clavicle or shoulder views may partially account for the discrepancy in performance seen between the AI models.

### Workflow implications

AI systems have the potential to optimise clinical workflow in multiple ways. There are several potential options: (1) available to bedside clinicians immediately following acquisition and processing; (2) available only to radiologists at the time of reporting; (3) used to prioritise abnormal findings for urgent review by the radiologist or bedside clinician; or some combination of these. All have benefits and disadvantages.

The major benefit of AI is its ability to produce near instantaneous interpretation of radiographs, which is not constrained by traditional working hours, not subject to recall payments, or susceptible to human fallibilities such as fatigue or hunger. Restricting AI output only to radiologists therefore limits much of its usefulness to emergency/trauma clinicians in providing timely decision making support. Previously expressed arguments for restricting access to AI only to radiologists to reduce the impact of false positives or negatives on patient management are less convincing when AI is operating similar to the level of a practising radiologist, as demonstrated here. High specificity of AI reported chest radiographs suggests it may be a useful adjunct when abnormalities are detected. However, for all pathologies, the absence of abnormality on AI did not reliably exclude it.

In four cases (0.3%), the AI was unable to identify chest radiographs as being radiographs and failed to produce an output. Technical limitations such as this highlight the role of AI as a diagnostic aid, rather than a radiologist replacement.

### Novel aspects of study

Here, we used contemporaneous CT to establish ground truth for a variety of trauma related findings. Most existing studies of radiograph interpretation use consensus subspecialist radiologist interpretation of the same image as the ground truth, which is likely to be less objective.^19,22^

In addition to the ground truth label, we compared AI against the report issued at the time by a general radiologist. Most retrospective studies compare AI against senior radiologist readers who know they are part of a research study, and this may overestimate true performance of radiologists in clinical practice. In this study, we compared the AI output to a different, and possibly slightly lower benchmark. This benchmark was the real radiology report produced by a general radiologist that was given to the bedside clinicians under real world reporting conditions. This is possibly a more fair comparison of the potential impact of AI in actual clinical settings.

Given the lack of large datasets, many previous AI studies have performed case-control studies with artificially inflated incidence of pathology.^15,21–24^ These datasets are not representative of real-world prevalence and meaningful interpretation can be difficult.^25,26^ Here we had access to a large cohort of patients with a consistent presentation (blunt trauma) but of varying severity and distribution of injury. This enabled us to perform a diagnostic accuracy study rather than a case-control study, providing stronger evidence of efficacy.

### Limitations

Although performed soon after one another, radiograph and CT were not performed simultaneously. Certain interventions may have been performed in the interval, *e.g.* insertion of an intercostal catheter which could affect the presence/absence pneumothorax on CT. However, the time between chest X-ray and CT was short (median 1.4 h and all were performed within 3 h). Therefore, the significant benefit of using CT with its increased specificity and sensitivity compared to chest radiograph likely outweighs this limitation.

In addition, in time-pressured real world reporting where radiologists know that CT is pending and will be the definitive diagnostic tool, radiograph reports may be shorter and focus on urgent clinical findings.

This study was limited to those situations where the radiograph was reported prior to CT acquisition. This likely excluded the majority of major traumas where there is usually a shorter time before CT, as well as excluding most traumas occurring out of normal working hours where due to limited staff reporting of cross-sectional imaging is prioritised, and radiographs are rarely reported first.

Finally as this was a retrospective study we do not know what information or other imaging the radiologist had access to at the time of reporting the chest radiograph. Radiologist’s superior ability to detect shoulder and humerus fractures may be a result of them having access to additional views or history when reporting.

### Further research

Further research should be done to assess the accuracy of Annalise.ai in non-trauma settings and to assess its ease of integration into existing clinical workflow. We encourage others to perform external validation studies of AI programs to ensure advertised accuracy is reflected in clinical practice. As AI performance is firmly established in the literature as achieving or exceeding radiologist interpretation, the focus of AI performance should shift towards comparing performance against a gold standard such as CT.

## Conclusion

This study showed that in the real-world trauma setting, the Annalise.ai algorithm provides timely reports with diagnostic accuracy at a comparable level to radiologists. In the short term, AI may provide a promising clinical decision support tool working as a hybrid model with radiologists. Prospective validation of these findings in the clinical setting is indicated.
